# Dysfunctional Behavioral Modulation of Corticostriatal Communication in the R6/2 Mouse Model of Huntington’s Disease

**DOI:** 10.1371/journal.pone.0047026

**Published:** 2012-10-04

**Authors:** S. Lee Hong, Desirée Cossyleon, Wajeeha A. Hussain, Lauren J. Walker, Scott J. Barton, George V. Rebec

**Affiliations:** 1 Program in Neuroscience, Indiana University, Bloomington, Indiana, United States of America; 2 Department of Kinesiology, Indiana University, Bloomington, Indiana, United States of America; 3 Department of Psychological and Brain Sciences, Indiana University, Bloomington, Indiana, United States of America; University of Florida, United States of America

## Abstract

**Background:**

In Huntington’s disease (HD), motor symptoms develop prior to the widespread loss of neurons in striatum and cerebral cortex. The aim of this study was to examine dysfunctional patterns of corticostriatal communication during spontaneously occurring behaviors in a transgenic mouse model of HD.

**Methodology/Principal Findings:**

Local field potentials (LFPs) were recorded from two closely interconnected areas, motor cortex and dorsal striatum, in wild-type controls (WT, *n* = 14) and a widely used transgenic HD model (R6/2 mice, *n = *12). All mice were between the ages of 7–9 weeks, a critical period of motor symptom development in R6/2s. Recordings were obtained while the mice were behaving freely in an open field. Specific LFP activity was extracted using timestamps for three increasingly demanding motor behaviors: 1) resting; 2) grooming; and 3) active exploration. Power spectral densities (PSD) were obtained for the cortical and striatal LFPs as well as coherence levels and relative phase across the frequency spectrum. In both brain regions, only R6/2s showed high frequency LFP oscillations during rest and grooming. As behavior increased from resting to exploring, corticostriatal synchrony at high frequencies declined in R6/2s, completely opposite to the WT pattern. R6/2s also exhibited nearly in-phase corticostriatal activity (cortex phase leads of ∼5°), while the WTs consistently showed cortical phase lags of ∼20° across all assessed behaviors, indicating a lead role for striatum.

**Conclusions/Significance:**

Our results add to growing evidence for altered communication between cortex and striatum in HD and suggest more generally that increasingly demanding motor behaviors differentially modulate corticostriatal communication. Our data also suggest conduction delays in R6/2 corticostriatal transmission, leading to compensatory speeding of LFP activity, as evidenced by the presence of high frequency LFP oscillations.

## Introduction

In Huntington’s disease (HD), a fatally inherited neurological condition, motor control deteriorates along with cortical and striatal neurons [1]. Long before these neurons die, however, they become dysfunctional [2], [3], suggesting that altered information flow through corticostriatal circuitry sets the stage for HD and its subsequent progression. In support of this view, both cortical and striatal neurons show aberrant patterns of spike activity in behaving transgenic mice that model HD [4], [5]. Relating the spike trains of individual neurons to spontaneously occurring behavioral events, however, is difficult because cortex and striatum modulate behavior through the oscillatory activity of large numbers of neurons. This local network activity, recorded as local field potentials (LFPs), may provide more relevant information about neuronal modulation of behavioral events than individual spikes [6]–[8]. In fact, oscillations in cortical and striatal LFPs have been implicated in goal-directed and spontaneous movement [9]–[13]. Our own research [14] has shown that, as behavior becomes more predictable, striatal LFP activity becomes less so. This is evident in our assessment of R6/2 mice, a widely used HD model, exploring a plus maze. The decreased tendency of these mice to turn left or right from the center choice point was associated with increased unpredictability of the striatal LFP signals.

Striatal neurons are driven, in large part, by cortical input, but also regulate cortical drive via multiple downstream connections [15], [16]. Here, we focused on two closely interacting regions, primary motor cortex and dorsal striatum. We recorded from both sites simultaneously and assessed LFP activity in R6/2 and aged-matched wild-type (WT) littermates behaving in an open-field environment. For analysis of LFP activity, we selected three behaviors that demanded increasing levels of motor complexity: 1) resting, which places no motor demands on the limbs; 2) grooming, which requires stereotyped patterns of forelimb movement; and 3) active exploration, which involves all the limbs in bouts of rearing and climbing. Power spectral densities (PSD) were assessed for each brain region. To determine how HD alters corticostriatal communication, we used coherence analysis to analyze LFPs for level of synchrony and relative phase. We found that, relative to WT, cortical and striatal LFPs in R6/2 mice possess a broader PSD distribution at higher frequencies, consistent with higher signal unpredictability. R6/2 mice also show deficits in corticostriatal communication that were reflected in the behavior-related distribution of coherence levels and the pattern of modulation of LFP synchrony.

## Results

**Figure 1:**
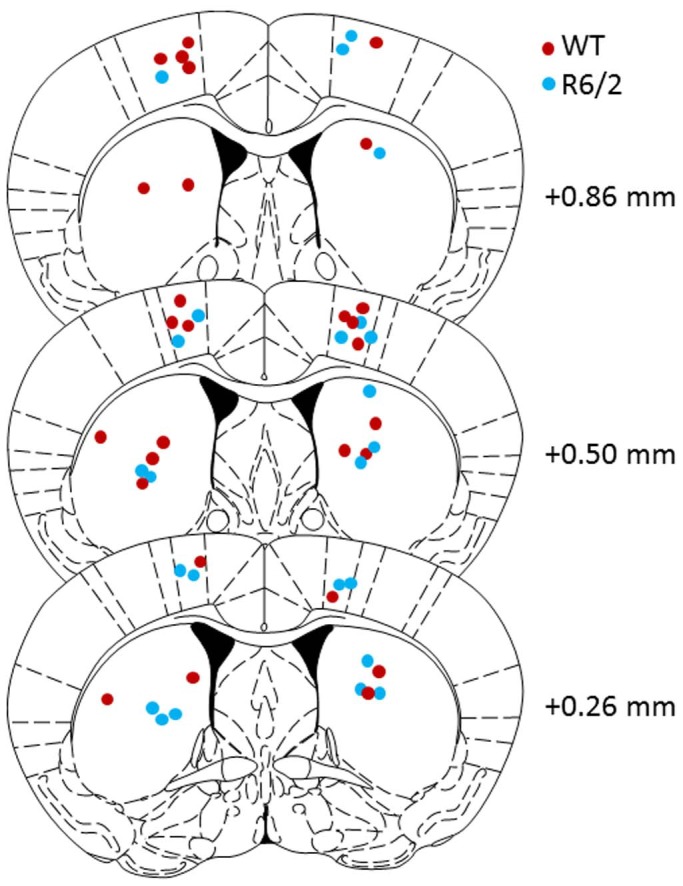
Histological analysis of electrophysiological recording sites. Schematic coronal sections of the mouse brain showing electrode placements in primary motor (M1) cortex and dorsal striatum of R6/2 (blue circles) and WT (red circles) mice. Numbers indicate distance anterior to bregma.

Data were collected when mice were 7–9 weeks old, a critical period of motor symptom development in R6/2s [17]. The mice were placed in an open field for a period of 30 min, while cortical and striatal LFP data were obtained through surgically implanted microelectrodes. Results of the histological analysis showed that electrodes were accurately placed within primary (M1) motor cortex and mid- to lateral dorsal striatum (Figure 1). This allowed the direct tracking of behaviorally relevant LFP activity; data were time-stamped to mark the initiation and termination of behavioral episodes for frequency analysis.

### Behavioral Analysis

**Figure 2:**
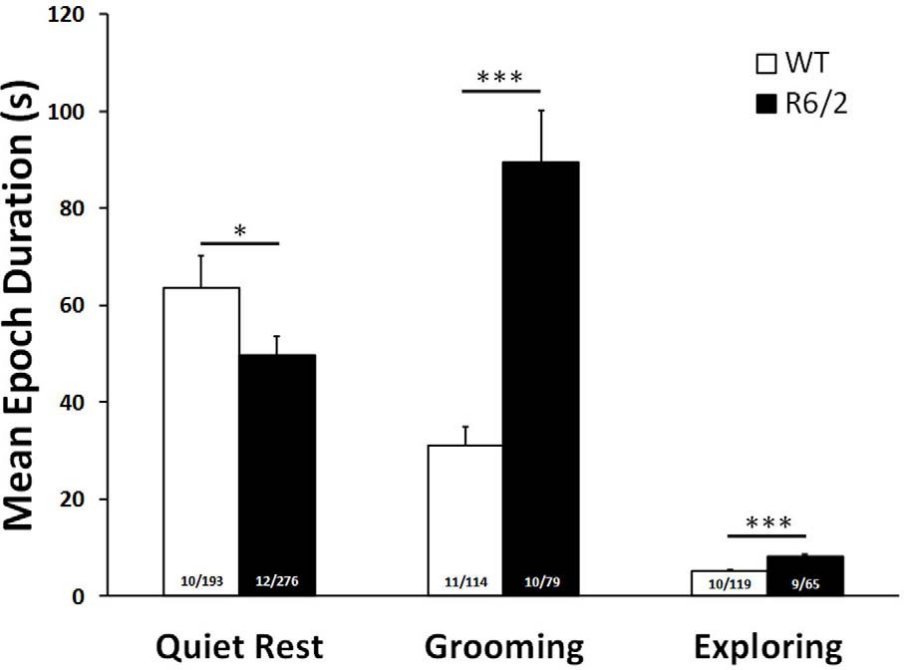
Mean behavioral epoch duration as a function of group and behavior. R6/2 mice engaged in grooming and active exploration for significantly longer periods than WT counterparts. Numbers in each column indicate the number of mice/epoch for each group at each behavior. * *p* = 0.05; *** *p*<0.001. Error bars denote SEM.

Behavioral data were obtained from 12 R6/2 and 14 WT mice. Although the number of instances of each assessed behavior was not significantly different between groups, R6/2 mice spent significantly more time grooming (*t*_(1,191)_ = 5.85; *p*<0.001) and exploring (*t*_(1,182)_ = 5.50; *p*<0.001) and less time in quiet rest (*t*_(1,467)_ = 1.96; *p* = 0.05) than WT mice. Significant group differences in duration of behavior are summarized in Figure 2. The number of mice displaying each behavior for analysis is indicated along with the number of instances of each behavior; some mice in each group did not engage in each behavior.

### LFP Frequency Spectra

**Figure 3:**
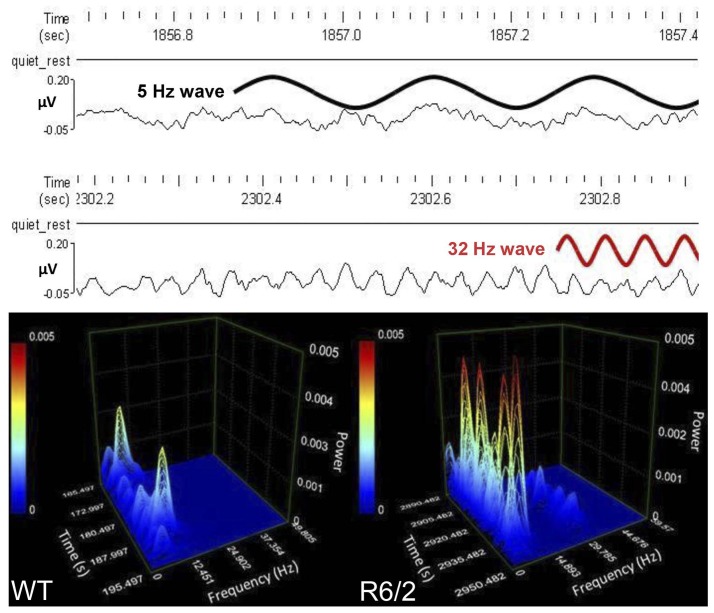
Exemplar raw LFP data from WT (black wave) and R6/2 (red wave) dorsal striatum and accompanying power spectral density (PSD) during quiet rest. Upper panel shows the raw LFP time series with accompanying waveforms that illustrate the strong presence of WT oscillations at low frequencies of ∼5 Hz (top) and characteristic ∼32 Hz (bottom) oscillations in R6/2. Lower panels show the accompanying PSD results as a time-frequency plot. The low-gamma oscillation at ∼32 Hz is characteristic of R6/2 LFPs and persists over time during quiet rest, but is not present in WT.

**Figure 4:**
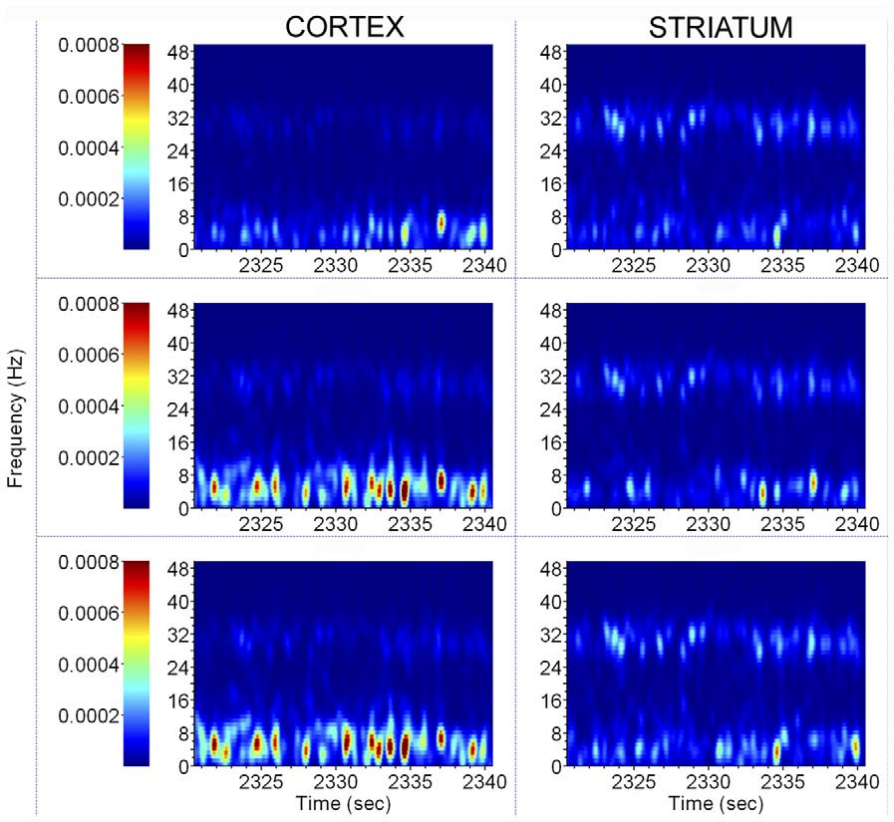
Exemplar PSD results from an R6/2 mouse during three different bouts of quiet rest. Time-frequency plots illustrate PSD obtained from motor cortex (left panels) and dorsal striatum (right panels) during three different bouts of quiet rest from an R6/2 mouse. The persistence of high frequency (∼32 Hz) rhythms is apparent in both brain regions with greater spectral power in striatum.

Since each mouse engaged in multiple episodes of a given behavior over the 30 min testing period with episodes of widely varying lengths, we calculated an average power spectrum for each animal for each behavior. This restricts analysis to group-level comparisons, which are necessary to minimize the effects of variance in behavioral episode duration on PSD estimates. Exemplar plots comparing WT and R6/2 LFP activity during quiet rest are presented in Figures 3 and 4. Highlighted in Figure 3 is the persistent presence of high frequency (∼32 Hz) oscillations in the striatum of R6/2 mice. High frequency oscillations within the same frequency range are also evident in cortex during quiet rest, albeit with less spectral power compared to striatum (Figure 4).

**Figure 5:**
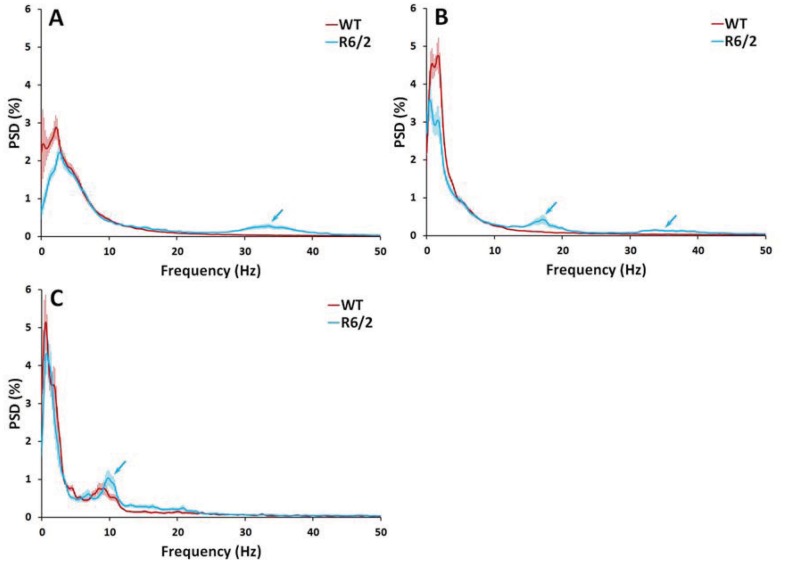
Striatal PSD across the different behaviors. Mean PSD curves obtained from striatal LFPs are denoted as a proportion of total power (% PSD) for WT (red) and R6/2 mice (blue) across quiet rest (A), grooming (B), and exploration (C). Error bars denote SEM around the group mean % PSD at each frequency. Arrows mark the higher frequency peaks in the R6/2 mice during resting and grooming as well as the 8–12 Hz theta band activity in both WT and R6/2 mice during exploration.

Although both groups engaged in many epochs of quiet rest, grooming, and exploration, there were clear behaviorally dependent differences in LFP frequency spectra between the groups. In both motor cortex and striatum, WT LFPs were dominated by low frequency oscillations across all assessed behaviors, whereas the R6/2 signal included high frequency oscillations during resting and grooming, despite resembling the WT pattern during exploration overall. These high frequency oscillations, shown for striatum and marked by arrows in Figure 5A–C, are present in R6/2s during all three behaviors, but are most distinctly different from WT during resting and grooming. Effectively, R6/2 corticostriatal activity was most disrupted when the behaviors placed lower demands on the motor system.

### Coherence Analysis

**Figure 6:**
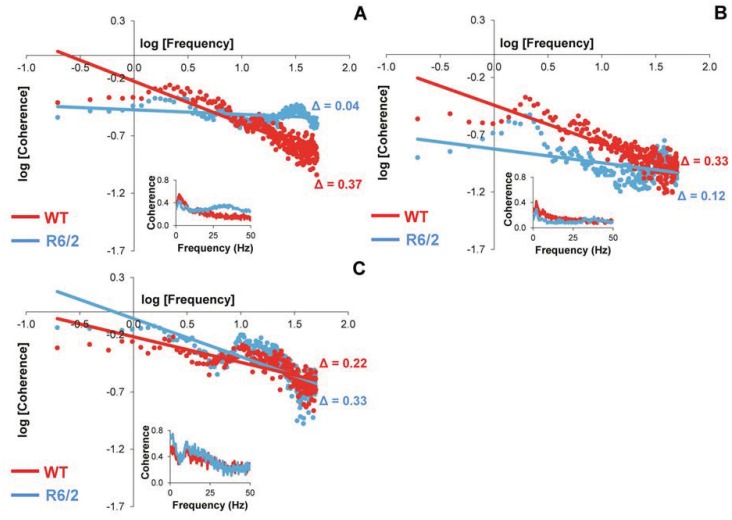
Corticostriatal coherence distributions. Log-log plots show the mean coherence spectrum across the frequency range from WT (red circles) and R6/2 (blue circles) mice during quiet rest (A), grooming (B), and quiet exploration. The correspondingly colored lines denote the least-squares fits to the coherence data. Slope values (Δ) for the fitted functions are provided adjacent to the lines of best fit in similar colored font. Data in their original linear coordinates are presented as insets in each panel.

We also analyzed corticostriatal coherence across the frequency spectrum to detect levels of synchrony between motor cortex and dorsal striatum. Because the two groups of mice exhibited clear differences in power spectra, comparing levels of synchrony within experimenter-defined frequency bands would not have provided a fair between-group comparison. As an alternative, we generated a summary statistic to capture the entire distribution of coherence levels across the power spectrum. To achieve this, we fitted a power law function to the mean coherence spectrum for each group and behavior. This approach allowed us to determine the rate of decay in coherence as a function of increasing frequency using the exponent value from each power law. The line of best fit was determined through linear least-squares regression in double-logarithmic space. The slope values in WT mice decreased with increasing behavioral activity, but R6/2s exhibited a completely opposite pattern (Figure 6A–C).

**Figure 7:**
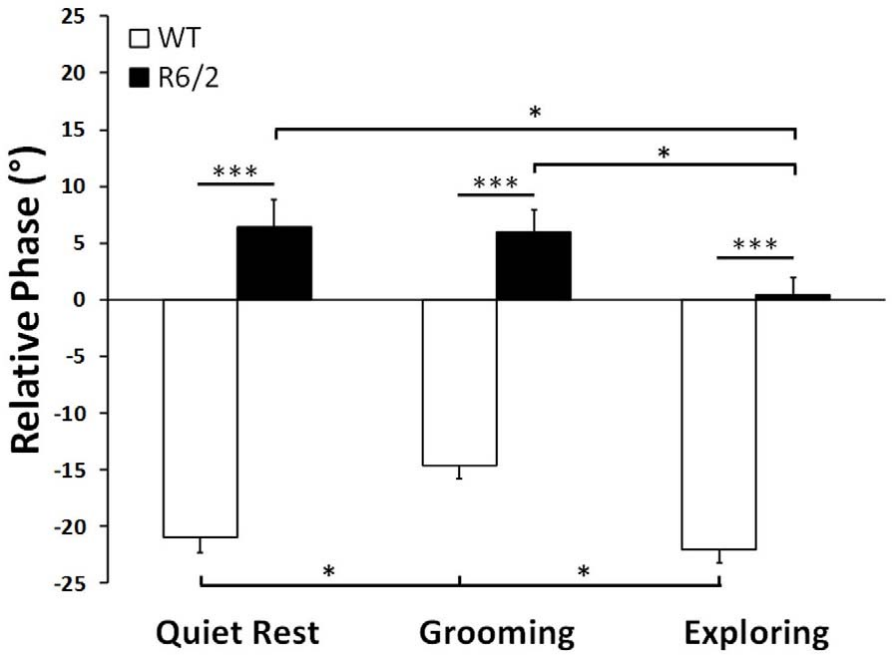
Mean relative phase for both groups across the different behaviors. Data represent means across the entire frequency range and animals within a group. For each column, the number of mice/epoch for each group at each behavior is the same as in Figure 2. * *p* = 0.05; *** *p*<0.001. Error bars denote SEM.

We also computed the relative phase between cortex and striatum from the PSDs. This analysis provides phase leads and lags from cortex to striatum. Analysis of the mean relative phase value across the frequency spectrum revealed significant group differences in relative phase (*F*_(1,1530)_ = 303.8; *p*<0.001), presented in Figure 7. The R6/2 cortical and striatal LFPs were almost perfectly in phase, showing very slight cortical phase leads of ∼5° during quiet rest and grooming. WT mice, in contrast, exhibited a phase lag of 15–20° across all assessed behaviors, indicating a lead role for striatum. Tukey-pairwise differences in relative phase between R6/2 and WT were noted for all three behaviors (*p*<0.001). Within genotype, the WT phase lag for grooming was significantly different (*p*<0.05) from both quiet rest and active exploration, whereas for R6/2s, active exploration was significantly different (*p*<0.05) from quiet rest and grooming. The group × behavior interaction was not significant.

## Discussion

Despite the onset of motor symptoms, R6/2 mice engaged in a similar number of episodes of all three behaviors (i.e., resting, grooming, and exploring). These mice, however, spent significantly more time grooming and exploring, and less time resting than the WT controls (Figure 2). Across each of these behaviors, markedly different corticostriatal LFP activity was evident in the high frequency activity and peaks that appeared consistently in the power spectra of R6/2 but not WT mice. The primary distinction between these groups of mice was the breadth of the distribution of the power spectrum at the low (<10 Hz) frequencies. Overall, the general “shape” of the LFP power spectra of both groups possessed similar characteristics, but the power spectra of R6/2 mice were visibly shifted rightward (toward higher frequencies) from that of WT mice. Furthermore, low frequency neural activity in R6/2s was distributed over a broader range of frequencies than WTs, when compared across similar behaviors.

At higher frequencies, we observed two principal features of R6/2 corticostriatal activity that were not present in WT: 1) the presence of high frequency activity around the low gamma (25–40 Hz) range that was especially prevalent in R6/2 striatum when the mice were at rest; and 2) beta (15–20 Hz) band activity in R6/2 mice during grooming (Figure 5B). Interestingly, gamma activity dissipated as the R6/2 mice increased their sensorimotor engagement with their environment, from resting to grooming. Similarly, beta activity in the R6/2 mice dissipated from grooming to exploring. The presence of high frequency LFP activity in the gamma and beta bands was not observed in the WT mice during rest and grooming, respectively.

From the viewpoint of distinct brain rhythms within set frequency bands, our data suggest that R6/2 mice exhibit aberrant electrophysiological activation not normally needed during a given behavior. During grooming, the presence of beta rhythms could indicate the need to stabilize the “motor program” by suppressing unnecessary movements [18], which would be akin to choreatic movements observed in humans with HD. Although we did not test for anxiety-like behavior, one possibility, based on the literature, is that the presence of gamma rhythms in R6/2 mice during rest can be linked to increased anxiety. Gamma rhythms have been shown to increase when worry is induced in people with generalized anxiety disorder [19]. This result is consistent with findings of anxiety-like behaviors previously observed in R6/2mice [20]. Alternatively, gamma rhythms during rest could be a reflection of motor problems. In monkeys, gamma rhythms measured from LFPs in motor cortex occur during shifts from quiet sitting to active engagement in reaching and grasping tasks [21]. This evidence suggests the intriguing possibility that there are ongoing motor-related neural oscillations in R6/2 mice even when they are at rest. Interestingly, major changes in hippocampal theta oscillations also occur during rest in HD mice, which may contribute to cognitive deficits [22]. Cortico-striatal gamma rhythms could potentially be a component of the uncontrollable choreatic movements characteristic of HD, in which brain activity normally reserved for voluntary motor function remains active during quiet rest. The precise role of gamma rhythms at rest in R6/2 mice, however, remains speculative, and the behavioral relevance of these rhythms should be evaluated in follow-up work.

The broader distribution of spectral power at higher frequencies in R6/2 mice is consistent with greater signal unpredictability, as we observed previously [14]. This change in power across the frequency range will result in greater signal entropy, which we found to be correlated with restricted behavioral flexibility in R6/2 mice [14]. Perhaps the presence of high frequency power in the R6/2 mice is a reflection of noise-contaminated cortical and striatal signals, with poorer quality information transmitted across the two regions. Distinguishing between signal and noise definitively, however, will require further research.

Interestingly, both groups exhibited a similar leftward shift in power spectral density as a function of increasing motor complexity (i.e., resting to grooming to exploring), resulting in a greater concentration of spectral power at low frequencies. As spectral power becomes concentrated at fewer frequencies, the signal becomes more predictable, as it comprises fewer oscillation rhythms. This is also consistent with our recent work showing similar patterns of adaptation of striatal LFP dynamics to behavioral decision-making in both R6/2 and WT mice [14]. Additionally, a clear peak in the theta (8–12 Hz) range was present for both R6/2 and WT mice during active exploration (consistent with Decoteau et al. [12]) that included rearing and climbing. This suggests that increased sensorimotor engagement reduces the differences between LFP rhythms between HD and controls.

The two groups of mice differed remarkably in the modulation of corticostriatal synchrony as a function of motor complexity (Figure 6). WTs exhibited an increase in relative contributions of synchrony at high frequencies (flattening slope) as a function of motor complexity (0.37 to 0.33 to 0.22). R6/2s showed the opposite, with a steepening slope (0.04 to 0.12 to 0.33), in which synchrony was increasingly concentrated at lower frequencies. Additionally, R6/2 mice exhibited a nearly flat slope during resting, indicating an almost equal distribution of synchrony across the frequency spectrum. Interestingly, functional magnetic resonance imaging of prodromal HD subjects evaluated at rest also showed dysfunctional corticostriatal connectivity [23].

Beyond altered patterns of corticostriatal synchrony, we also observed marked differences in relative phase across the coherence spectrum between the two groups. The R6/2 mice exhibited virtually in-phase (∼zero lag) activity across the corticostriatal coherence spectrum, while the WT mice exhibited a slight mean phase difference, with a cortical phase lag of ∼20° across all three behaviors (Figure 7). These group differences persisted across all behaviors, suggesting this difference in relative phase across the R6/2 and WT might be a component of the breakdown of corticostriatal communication. Interestingly, as per Fries [24], 20° is more than an order of magnitude shorter than a complete cycle, i.e., 360° and is consistent with naturally occurring conduction delays.

A slight striatal phase lead seen in WT mice is consistent with natural transmission delays arising from the spatial distance between the two brain regions. The near-zero phase lag and inverted corticostriatal lead-lag relationship in HD mice could be a reflection of conduction delays in which the transmission of neural information from striatum to cortex is slowed by approximately an entire cycle. Conduction delays due to HD provide another explanation for the presence of higher frequency LFP activity across all behaviors. The increased presence of high frequency LFP rhythms in HD could be a reflection of compensatory speeding of neural transmission in order to overcome conduction delays.

It appears, therefore, that the breakdown in communication between cortex and striatum alters the manner in which different levels of increasingly demanding motor patterns are achieved in HD. In addition, HD results in an in-phase relationship between cortex and striatum, suggestive of conduction delays in which corticostriatal communication has been slowed by almost an entire oscillation cycle. These results present an intriguing new finding that the effects of HD on corticostriatal synchrony are not necessarily revealed in the overall or average level of coherence across the frequency spectrum or within specific frequency bands or brain rhythms. Instead, the distribution of corticostriatal coherence across the frequency spectrum provides important insight into the difference between neural patterns in HD and controls. Furthermore, HD-related deficits in patterns of corticostriatal communication can be revealed through aberrant behavior-dependent modulation of the distribution of coherence across the frequency spectrum.

### Conclusions

Our results support the hypothesis that high levels of neural signal unpredictability precede neuron death in HD, as evidenced by the broader frequency spectra in R6/2 mice. Specifically, we identified the following three possible sources of high frequency activity in LFP activity in motor cortex and dorsal striatum: 1) motor program instability (beta rhythms) and motor- or possibly anxiety-related complications (gamma rhythms); 2) neural noise; and 3) corticostriatal conduction delays. Accompanying the high frequency activity in cortical and striatal rhythms, our data also provide evidence of dysfunctional corticostriatal communication in HD. The differences between R6/2 and WT mice are especially apparent when examined with respect to behaviorally relevant modulation of neural activity. In fact, HD mice exhibited a completely different pattern of change in corticostriatal communication as a function of behavioral complexity in comparison to WT controls. These data suggest that declining flexibility in behavior in models of HD [14] is associated with dysfunctional communication between cortex and striatum. It will be interesting to determine if this pattern of neurobehavioral dysfunction is specific to HD or is a common occurrence across a variety of different neurodegenerative disorders, indicative of impending widespread neuron loss.

## Methods

### Ethics Statement

All efforts were made to minimize the number of animals used and their suffering, and certify that animal use followed guides approved by the Institutional Animal Care and Use Committee of Indiana University. These guidelines were established by the National Institutes of Health Guide for the Care and Use of Laboratory Animals (NIH Publications No. 80–23, revised in 1996). The present study was specifically approved by the Bloomington Institutional Animal Care and Use Committee.

### Animals

Data were obtained from 12, male R6/2 mice (B6CBA-TgN[HDexon1]62Gpb), which express an expanded CAG repeat in exon 1 of the human HD gene, and 14, male WT littermate controls. All mice were obtained from the Jackson Laboratories (Bar Harbor, ME) at 5–6 weeks of age and housed individually in the department animal colony under standard conditions (12-hr light-dark cycle with lights on at 07∶30) with food and water provided ad libitum. Testing began 2–3 weeks (i.e., at ages of 7–9 weeks) after arrival when R6/2s express early but robust neurological signs [25].

### Genotype and CAG Repeat Length

The genotype of each subject animal and CAG repeat length in R6/2 mice were determined from tail tissue samples with PCR and subsequent analytical agar’s gel electrophoresis as previously described [4]. Gels were evaluated with Kodak Image Station 4000R and Kodak Molecular Imaging software (Carestream Molecular Imaging, New Haven, CT). Using Clone Manager software (Sci-Ed Software, Cary, NC), we aligned primers to exon 1 of the *huntingtin* gene sequence acquired from the National Center for Biotechnology Information (http://www.ncbi.nlm.nih.gov). Alignment of primers to template indicated that the R6/2 DNA fragment amplified by PCR is 104 bp longer than the CAG repeat region. Computer analysis of fragment migration against a 100-base pair DNA standard ladder showed that our experimental R6/2 mice had 122.0±1.4 (mean ± SE) repeated CAG codons.

### Surgery

After approximately one week of habituation to the colony, mice were anesthetized with a mixture of chloral hydrate and sodium pentobarbital (chloropent, 0.4 ml/100 g, ip) and secured in a stereotaxic frame in preparation for subsequent electrophysiological recording as described previously [4]. A unilateral hole was drilled +0.5 mm anterior and ±1.5 mm lateral to bregma, according to standard coordinates [26]. Multi-wire electrode bundles were lowered into M1 cortex and dorsal striatum (0.6 mm and 3.0 mm ventral to brain surface, respectively). Two, stainless steel anchor screws and dental acrylic ensured permanent attachment of the electrode assembly to the skull. Antibiotic cream was applied to the surgical site to prevent infection. Lactated Ringer solution (1 ml, sc) was administered to counteract dehydration. All mice were allowed one week of postsurgical recovery, during which they were monitored closely to ensure a healthy recovery.

### Behavioral Electrophysiology and Time-Stamping

Electrode bundles were constructed in-house; each consisted of four, 25 µm (diameter) Formvar-insulated stainless steel wires and one 50 µm uninsulated stainless steel ground wire. Bundles were friction fitted to gold pin connectors in a custom polyphenylene sulfide hub (7×6×4 mm) (Omnetics Connector Corporation, Minneapolis, MN, USA). On the recording day, the electrode assembly was connected to a lightweight flexible wire harness equipped with field-effect transistors that provided unity gain current amplification for each wire. LFPs were routed through preamplifiers with 1000× gain and 0.7–170 Hz filters. Neural signals were digitized at 40 kHz and acquired by a multichannel acquisition processor (Plexon, Dallas, TX, USA).

The open field (25×18 cm with outwardly angled walls 17 cm high) was placed in a sound-attenuating and electrically shielded recording chamber. The harness was connected to a swiveling commutator, allowing the mice to behave freely. After a 5–10 min habituation period, data were collected for 30 min. Open-field behavior was videotaped, time-stamped, and synchronized with electrophysiological recording. Videotapes were later reviewed and coded by independent observers blind to genotype. Each behavioral episode was grouped into three categories for analysis: 1) quiet rest; 2) grooming; and 3) exploration. Quiet rest was defined as the absence of overt movement. Grooming referred to stereotyped face washing or fore- or hindlimb scratching. Exploration included episodes of rearing and/or climbing up the sloped open field walls. *Note that bouts of locomotor activity (i.e., walking and running) were not included in exploration, as distinct start and stop time points of this behavior could not be accurately defined through visual observation.* Only behavior within each category lasting ≥3.0 s was extracted for analysis.

### Data Analysis

NeuroExplorer (NEX; Littleton, MA, USA) was used to mark the start and stop time of each behavioral episode and to analyze corresponding LFPs. NEX also was used in conjunction with custom-written MATLAB scripts (Mathworks, Natick, MA, USA) to generate power spectral density (PSD) data for M1 cortical and striatal recording electrodes in each mouse using a Fast Fourier Transform across the frequency range of 0 to 50 Hz with intervals of 0.195 Hz. To allow for level comparisons across different conditions and groups, the PSDs were normalized and represented as a percentage of total spectral power. PSDs obtained for individual behavioral epochs were averaged across repeated engagements in the same behavior. This was done to minimize the effects of the heterogeneity of duration and frequency of the behavioral epochs on the frequency analysis. PSD data were then averaged across electrodes, yielding a PSD for each behavior for every mouse.

Further analysis was performed using the cross power spectra from cortex and striatum, allowing us to obtain coherence and relative phase values across the frequency range. Coherence values ranged from 0 to 1, where a value of 1 represents a perfect synchrony between the cortex and striatal signals for a given frequency. Relative phase values ranged from −180° to 180°, where zero relative phase indicates an in-phase corticostriatal relationship and 180° indicates an anti-phase pattern. Because the cortex was used as the reference signal, positive relative phase values indicate cortical phase leads while negative values indicate striatal phase leads (or cortical phase lags). To obtain the rate of decay in corticostriatal coherence as a function of increasing frequency, we obtained the exponent of a power law function through a double-logarithmic transformation of the data, such that *y  =  ax^b^* is transformed to log *y  =  log a + b log x.*

Statistical analyses used GraphPad Prism 5 (GraphPad Software, San Diego, CA, USA) with the α-level for significance set at *p*<0.05. Two-tailed independent samples *t-*tests were used to compare group differences in duration and frequency of each behavior. A group × behavior ANOVA was conducted on the relative phase values of the power spectra.

### Histology

To verify electrode placement after completion of all recording sessions, mice were sacrificed with an overdose of chloropent (> twice the surgical dose) and a current pulse (30 µA for 10 s) was passed through each active microwire to mark recording sites. Mice were then transcardially perfused with saline followed by 10% potassium ferrocyanide [K_4_Fe(CN)_6_] in 10% paraformaldehyde to produce small blue deposits at the site of the recording electrode (“Prussian blue” reaction). Brains were removed, post-fixed in 10% paraformaldehyde for one hour, and cryoprotected in 30% phosphate-buffered sucrose. The brains were then frozen; coronal sections (60 µm) were then cut on a sliding microtome and mounted on gelatin-subbed slides. The sections were stained with cresyl violet and examined under a light microscope to confirm microwire location. Only recordings with clear electrode placements in M1 cortex and dorsal striatum were used for analysis.
